# Ovicidal and Larvicidal Effects of *Piper marginatum *Essential Oil on *Lutzomyia longipalpis* (Lutz & Neiva, 1912)

**DOI:** 10.1007/s13744-026-01422-6

**Published:** 2026-08-19

**Authors:** Lucas Alexandre Farias de Souza, Michele Trombin de Souza, Mireli Trombin de Souza, Damaso Rubio-Vargas, João Carlos Minozzo, Beatriz Helena Lameiro de Noronha Sales Maia, Magda Clara Vieira da Costa-Ribeiro

**Affiliations:** 1 Vector Insects and Parasites Lab, Dept of Basic Pathology, Federal Univ of Paraná, Curitiba, Paraná Brazil; 2 Dept of Cellular Biology, Federal Univ of Paraná, Curitiba, Paraná Brazil; 3 Center for Production and Research of Immunobiologicals, Curitiba, Paraná Brazil; 4 Dept of Chemistry, Federal Univ of Paraná, Curitiba, Paraná Brazil

**Keywords:** *Lutzomyia longipalpis*, Sand fly, *Leishmania infantum*, Visceral leishmaniasis, Eggs, Larvae, Control

## Abstract

*Lutzomyia longipalpis* (Lutz & Neiva, 1912) is the main vector of the protozoan *Leishmania infantum*, the etiologic agent of Visceral Leishmaniasis (VL) in the American continent. In Brazil, the control of this sand fly species is difficult due to its resistance to chemical insecticides. The present study had the objectives of determining the chemical composition of *Piper marginatum* essential oil (EO) and to evaluating its ovicidal and larvicidal activity against *Lu. longipalpis* under laboratory conditions. To achieve this, a gas chromatography-mass spectrometry (GC-MS) was performed, and nine concentrations of the EO were tested on eggs and larvae: 0.25%, 0.5%, 1%, 1.5%, 2%, 2.5%, 3%, 3.5%, and 4%. Probit analysis estimated LC₅₀ and LC₉₀ values of 1.18% and 2.41% for eggs, and 0.91% and 1.71% for larvae, respectively. The major constituent of *P. marginatum* EO was 3',4'-methylenedioxypropiophenone, a phenylpropanoid commonly found in *Piper* species. All concentrations higher than 1% killed more than 50% of the treated larvae and rendered more than 50% of the eggs unviable. This is the first report of the toxicity of EO from a *Piper* plant against a sand fly species. This EO may constitute a viable alternative for controlling the immature stages of the primary vector of VL.

## Introduction

In the American continent, Visceral Leishmaniasis (VL) is a serious public health problem that, if the adequate treatment is not promptly initiated, can progress to death (OPAS 2024). This chronic systemic disease is caused by the protozoan *Leishmania infantum,* which is transmitted through the bites of female sand flies, specifically *Lutzomyia longipalpis* (Lutz and Neiva, 1912) (Psychodidae: Phlebotominae), the main vector species on the continent (Lainson and Rangel 2005).

Since there is no vaccine for leishmaniasis, the main strategy available for reducing its incidence is vector control. In Brazil, a country with 91% of the VL cases that occur in Americas (OPAS 2024), the pyrethroid insecticide alpha cypermethrin has been used to control the *Lu. longipalpis* population in the residences (SVS 2014). For many years, this method has proven efficient (Alexander et al. 2009). However, a reduction in the sensitivity of *Lu. longipalpis* to this insecticide has been observed (Barata et al. 2011), compromising the vector control program adopted in Brazil.

In this context, screening of natural products has attracted the attention of researchers seeking new efficient methods for controlling sand flies. In recent years, the search for plant-derived insecticides has been increasingly intensified (Amane et al. 2023). These products have several advantages when compared to synthetic insecticides: they are obtained from renewable resources, degrade quickly and do not leave residues in food or the environment (Broussalis et al. 1999; Corrêa et al. 2011; Zoubiri et al. 2014; Demirak and Canpolat 2022).

Essential oils (EOs) are intricate blends of volatile organic compounds, commonly utilized in the food, pharmaceutical, and pest control industries. A variety of these oils possess diverse biological effects, including insecticidal, antibacterial, and antifungal properties (Tian et al. 2020). Some studies have shown the potential use of EOs of plant origin against *Lu. longipalpis* at different life stages (Maciel et al. 2010; Mota et al. 2022; Rosa et al. 2024). Using EOs to manage sand flies may offer an environmentally friendly option to minimize chemical use.

The genus *Piper* is primarily found in tropical and subtropical regions worldwide. It has been widely studied as a source of novel natural compounds with potential antifungal, antitumor, antioxidant, antiplasmodial, and trypanocidal activities (Lago et al. 2009). The species *Piper marginatum* Jacq. (Piperales: Piperaceae), described by Nikolaus Joseph von Jacquin in 1760 and popularly known as Pimenta do mato (Autran et al. 2009), is abundant in Brazilian territory and is used to treat gastrointestinal diseases, in addition to serving as a tonic, diuretic, and carminative (Bru and Guzman 2016). The EOs of *P. marginatum* have already been tested against several insect species of sundry taxonomic groups. For example, the oil showed activity against mosquitoes such as *Aedes aegypti* (Linnaeus) (Autran et al. 2009) and agricultural pests such *Planococcus citri* Risso (De Lima et al. 2024) and *Drosophila suzukii* Matsumura (de Souza et al. 2020).

Based on this, the present study had the objectives of determining the chemical composition of *P. marginatum* EO and evaluating its ovicidal and larvicidal activities against *Lu. longipalpis* under laboratory conditions.

## Material and methods

### Laboratory rearing of sand flies

The establishment and maintenance of the sand fly colony were based on the methods of Lawyer et al. (2017), with some modifications. The insects were kept in nylon tulle cages inside an incubator at 27°C and 80% relative humidity. The adult females were fed anesthetized mice (10 mg/kg of Ketamine, and 2 mg/kg of Xylazine administered intramuscularly). After 4 days, females were placed in plastic pots that were internally coated with sterile plaster to maintain moisture and filled with a substrate prepared with rabbit feces, rabbit food, fish food and soil. Afterward, the eggs were transferred to similar pots for hatching, and the hatched larvae were maintained on this substrate until pupation. When the adults emerged, they were transferred to cages to continue the cycle.

### Extraction and chemical characterization of essential oil

The extraction of EO was performed using the hydrodistillation method with a Clevenger-type apparatus. Dried leaves of *P. marginatum* collected in Novo Progresso, Brazil (S 7° 07′ 43.56″ W 55° 23′ 22.09″), were crushed and placed in a glass flask containing 1.0 L of distilled water, remaining in boiling for 4.5 h (h) for extraction. To determine the EO content on a dry basis, the total mass of each EO obtained was considered in relation to the dry mass of the botanical material used for extraction. After extraction, the samples were collected with a precision pipette and stored in a freezer until analysis.

The chemical constituents of the EO were identified by gas chromatography coupled with mass spectrometry (GC–MS). The oil was diluted to 1% concentration in dichloromethane, and 1.0 μL of the resulting solution was injected with a split ratio of 1:20 into an Agilent 6890 gas chromatograph (Agilent Technologies, Palo Alto, CA, USA) connected to an Agilent 5973 N mass selective detector. Electron ionization was performed at 70 eV, using a scanning rate of 3.15 s⁻^1^ over a mass range of 40-450 u. The transfer line, ion source, and quadrupole analyzer temperatures were maintained at 260°C, 230°C, and 150°C, respectively.

The identification of the chemical constituents was carried out by comparing their mass spectra with those from spectral libraries and by using their linear retention indices, calculated from the injection of a homologous series of hydrocarbons (C7-C26).

### Experimental design

The *P. marginatum* EO was emulsified according to de Souza et al. (2020). Briefly, Tween 80 served as the emulsifying agent at a fixed concentration of 0.5% (v/v) in all treatments. For each assay, the required volume of EO was first mixed with the surfactant to form a pre-emulsion, followed by gradual addition of distilled water under vigorous stirring until the final volume was reached. Using this procedure, EO concentrations ranged from 0.25% to 4.0% (v/v), whereas the emulsifier level remained unchanged throughout the experiments.

Nine EO concentrations were tested against eggs and larvae of *Lu. longipalpis*: 0.25% (2.5 mg/mL), 0.5% (5 mg/mL), 1% (10 mg/mL), 1.5% (15 mg/mL), 2% (20 mg/mL), 2.5% (25 mg/mL), 3% (30 mg/mL), 3.5% (35 mg/mL), and 4% (40 mg/mL). Distilled water and Tween 80 (0.5% v/v) were used as negative controls, while cypermethrin (0.196 mg/mL) as the positive control.

Five-day-old eggs and second-instar larvae (six days after hatching) were used in the bioassays. For each developmental stage, individuals were assigned to 12 experimental groups: nine corresponding to the EO concentrations tested and three serving as positive and negative controls. Each group consisted of ten individuals. Eggs and larvae were placed in the center of 50-mL plastic containers lined with a thin layer of plaster to maintain moisture. Subsequently, 1 mL of the respective treatment solution was sprayed onto each container using a hand-held sprayer. The experiment was conducted with ten replicates. Egg mortality was assessed daily for up to three days after EO application.

Lethal concentrations causing 50% and 90% mortality (LC₅₀ and LC₉₀) for eggs and larvae were estimated using Probit analysis based on concentration-mortality data at 72 h after exposure to treatments.

### Enzymatic activity

For the acetylcholinesterase (AChE) activity assay, larvae were exposed to the LC₅₀ and LC₉₀ concentrations of *P. marginatum* EO. Five independent pools, each containing 20 s-instar larvae, were prepared per treatment. Pools treated with distilled water, Tween 80, and cypermethrin were used as negatives and positive controls, respectively. After exposure, samples were homogenized in 0.1 M potassium phosphate buffer (pH 7.5) using a tissue lyser. Total protein concentration was determined by the Bradford method (Bradford 1976), and all samples were normalized to 1.0 mg protein/mL using the same buffer.

Acetylcholinesterase activity was determined in 96-well microplates following the method of Ellman et al. (1961). Briefly, 20 µL of the sample supernatant was added to each well (in triplicate), followed by 130 µL of DTNB solution and 50 µL of acetylthiocholine iodide as substrate. Control wells contained 20 µL of buffer instead of the sample. The increase in absorbance was measured immediately at 405 nm for 5–7 min, with readings taken at 40–52 s intervals, using a microplate spectrophotometer. Enzymatic activity was expressed relative to protein concentration. Each assay was conducted in quintuplicate.

### Statistical analysis

All statistical analyses were performed using GraphPad Prism version 8.3 (GraphPad Software, San Diego, CA, USA). Data were initially assessed for normality using the Shapiro–Wilk test. When data did not meet normality assumptions, nonparametric analyses were applied using the Kruskal–Wallis test followed by Dunn’s multiple comparisons test. For datasets that met normality assumptions, differences among treatments were analyzed by one-way analysis of variance (ANOVA), followed by Dunnett’s multiple comparisons test when comparisons with the negative control were required. Results are presented as median and interquartile range for nonparametric data or as mean ± standard deviation for parametric data. Differences were considered statistically significant at *p* < 0.05. Furthermore, to estimate the lethal concentrations (LC_50_ and LC_90_), a binomial model with a complementary log–log link function (Gompit model) was employed using the Probit Procedure in the software SAS version 9.2 (SAS Institute 2011).

## Results

### Chemical characterization of essential oil

Nineteen chemical components were identified in *P. marginatum* EO by GC–MS. They are listed in Table 1. The five most abundant compounds were 3',4'-methylenedioxypropiophenone (18.52%), gamma-terpinene (8.05%), beta-caryophyllene (8.49%), trans-ocimene (7.49%) and elemicin (6.07%).

**Table 1:** Relative percentage composition of essential oil of *Piper marginatum*

Nº	Constituents	[] %
1	alpha-pinene	2.15
2	beta-pinene	1.43
3	alpha-phellandrene	5.09
4	alpha-terpinene	3.09
5	p-cymene	4.26
6	cis-ocimene	4.85
7	trans-ocimene	7.49
8	gamma-terpinene	8.05
9	isoterpinolene	4.22
10	linalool	1.35
11	alpha-copaene	4.05
12	beta-caryophyllene	8.49
13	bicyclogermacrene	5.66
14	myristicin	4.75
15	delta-cadinene	1.63
16	3',4'-methylenedioxypropiophenone	18.52
17	elemicin	6.07
18	E-nerolidol	1.51
19	spathulenol	5.61

### Ovicidal effect

**Fig. 1:**
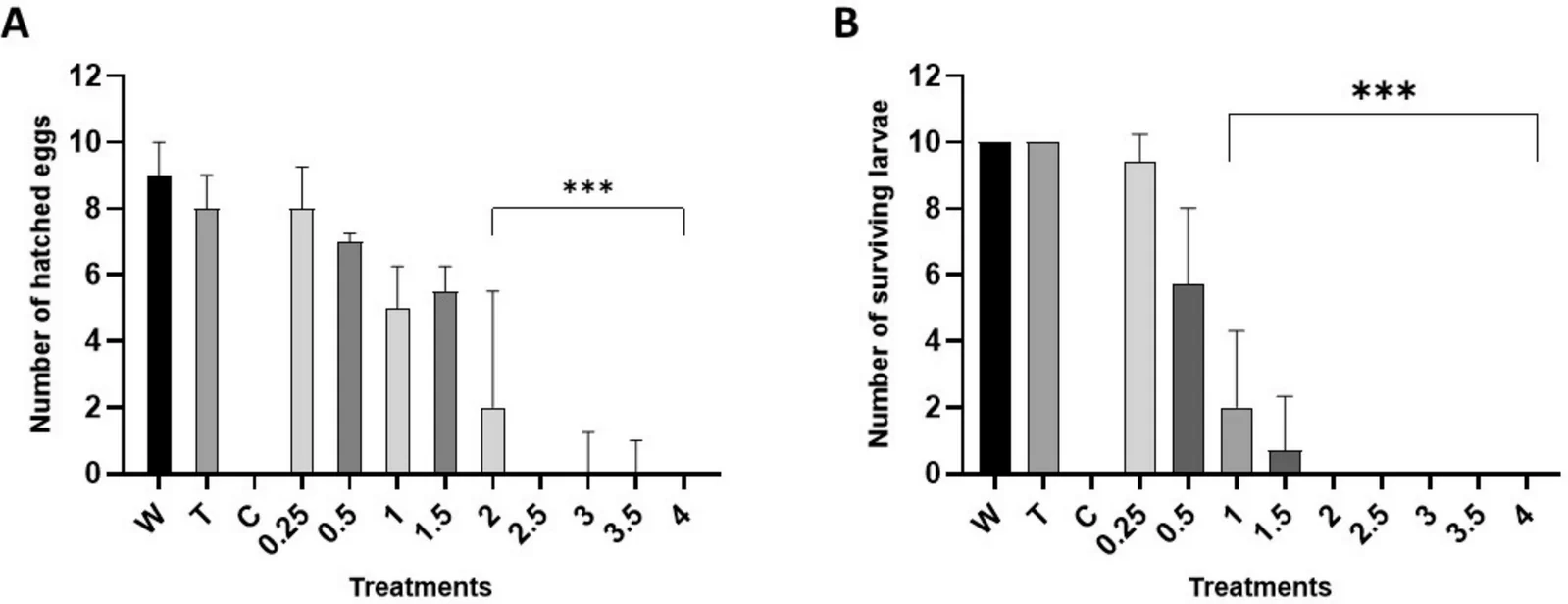
Ovicidal and larvicidal effects of *Piper marginatum* essential oil (EO) on *Lutzomyia longipalpis*. (**A**) Ovicidal activity expressed as the mean number of hatched eggs (± SD) after 72 h of exposure to increasing concentrations of the EO (0.25-4.0% v/v). (**B**) Larvicidal activity expressed as the mean number of surviving larvae (± SD) 72 h post-treatment. Water (W) and Tween 80 (T) were used as negative controls, and cypermethrin (C) as the positive control. Each treatment consisted of 10 independent replicates, with 10 individuals per replicate. Data were analyzed using the Kruskal–Wallis test followed by Dunn’s multiple comparison test. Comparisons were performed between each EO concentration and the negative control (distilled water). Asterisks (***) indicate statistically significant differences relative to the negative control (*p* < 0.001)

The effect of *P. marginatum* EO on the egg hatchability of *Lu. longipalpis* was statistically significant, as revealed by the Kruskal–Wallis’s test (H = 102.4, df = 11, *p* < 0.0001). All EO concentrations above 0.25% significantly reduced egg hatching compared with the negative control (water). At the lowest concentrations tested, 0.25% and 0.5%, the hatching rates were 84% and 63%, respectively. A more pronounced inhibition of egg hatching was observed at concentrations ≥ 1% (Kruskal–Wallis’s test followed by Dunn’s multiple comparison test, *p* < 0.05), with treatments ranging from 1.5% to 4% inhibiting more than 50% of egg hatchability. Complete inhibition of egg hatching was observed at the 4% concentration, similar to the positive control (cypermethrin). In the negative control groups, the hatching rates were 90% for distilled water and 83% for Tween 80 (Fig. 1A). Post hoc comparisons using Dunn’s test confirmed significant differences between treatments ≥ 1% and the negative controls. Based on the concentration–response curves and the overlapping confidence intervals of the LC_50_ and LC_90_ values, we found for eggs 1.18% (CI: 1.14-1.21%) and 2.41% (CI: 2.35-2.48%), respectively.

### Larvicidal effect

Regarding the larval stage, larval survival differed significantly among treatments, as indicated by the Kruskal–Wallis test (H = 108.3, df = 11, *p* < 0.0001). EO concentrations higher than 1% caused more than 50% larval mortality within 72 h post-application (Fig. 1B), whereas treatments ranging from 1.5% to 4% resulted in complete larval mortality. At the highest concentrations tested (3.5% and 4%), all larvae died within 4 h of exposure, while the positive control (cypermethrin) caused complete mortality within 3 h (Table 2). No larval mortality was observed in the water and Tween 80 control groups. Probit analysis estimated LC₅₀ and LC₉₀ values of 0.91% (CI: 0.71-1.11%) and 1.71% (CI: 1.46%-1.96%) for larvae, respectively.

**Table 2:** Mean of larval survival after treatments

	1 h	2 h		3 h	4 h	5 h	6 h	12 h	24 h	48 h	72 h
Cypermethrin (0,196 mg/ml)	9.4	6.1		0	0	0	0	0	0	0	0
Water	10	10		10	10	10	10	10	10	10	10
Tween 80	10	10		10	10	10	10	10	10	10	10
EO 0.25%	10	10		10	10	9.9	9.9	9.6	9.6	9.4	9.4
EO 0.5%	10	9.8		9.8	8.6	8.6	8.6	7.6	7.2	6.1	5.7
EO 1%*	10	9.8		7.4	3	3	3	2.4	2.4	2	2
EO 1.5%*	10	8.6		4.8	1.5	1.5	0.7	0.7	0.7	0.7	0.7
EO 2%*	10	9.5		3	0.5	0.5	0	0	0	0	0
EO 2.5%*	9.8	9.4		3.1	0.6	0.6	0.3	0.2	0.2	0	0
EO 3%*	9.4	8.5		2.7	0.4	0.4	0.4	0.2	0.2	0	0
EO 3.5%*	10	9.4		1.2	0	0	0	0	0	0	0
EO 4%*	9.5	8.5		0.4	0	0	0	0	0	0	0

Mean of surviving larvae after treatment with different concentrations of essential oil (EO). *Significant values ​​(*p* < 0.001) when compared with negative control using the Kruskal–Wallis test

### Enzymatic activity

**Fig. 2:**
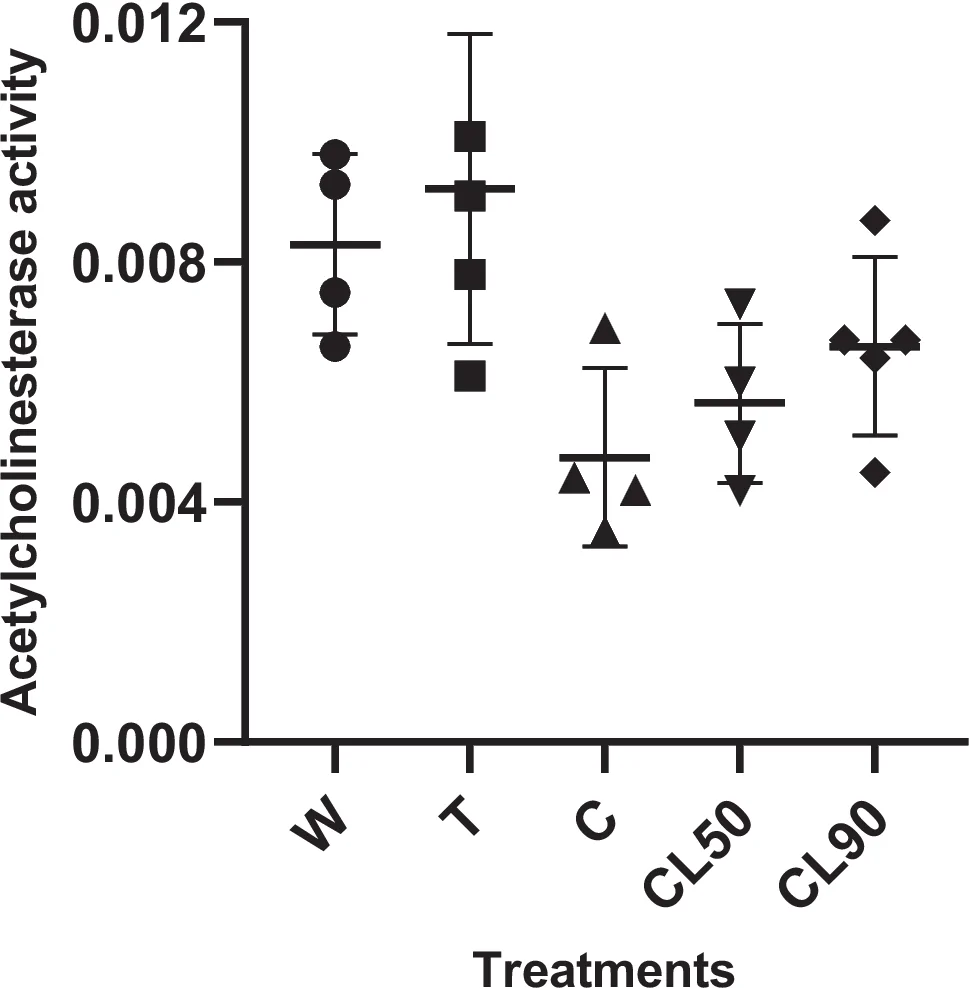
Acetylcholinesterase activity (µmol/min/mg protein) in *Lutzomyia longipalpis* larvae exposed to *Piper marginatum* essential oil (EO). Enzymatic activity was measured in larvae treated with LC₅₀ and LC₉₀ concentrations of the EO. Circles (●) water control (W), squares (■) Tween 80 control (T), upward triangles (▲) cypermethrin-treated group (C), downward triangles (▼) LC₅₀ group, and diamonds (◆) LC₉₀ group. Symbols represent individual biological replicates, and horizontal bars indicate mean ± SD. Data were analyzed using one-way ANOVA followed by Dunnett’s multiple comparison test, comparing each treatment with the negative control group

Acetylcholinesterase activity differed among treatments, as indicated by the one-way ANOVA (F₄,₁₇ = 4.65, *p* = 0.0101). A decreasing trend in enzyme activity was observed in larvae exposed to the LC₅₀ and LC₉₀ concentrations of *P. marginatum* EO when compared with the negative control. Mean enzyme activity values were 0.0092 µmol min⁻^1^ mg⁻^1^ protein for the Tween 80 group, 0.0083 for the water control, 0.0066 for LC₉₀, 0.0057 for LC₅₀, and 0.0047 for cypermethrin (Fig. 2). However, post hoc multiple comparisons did not reveal statistically significant differences between individual treatment groups.

## Discussion

This study provides the first evidence of the toxic effects of an EO derived from a *Piper* species on the eggs and larvae of sand fly. The chemical composition of *P. marginatum* EO was characterized, and its ovicidal activity was assessed based on egg hatching inhibition, as well as its larvicidal activity against second-instar *Lu. longipalpis* larvae. In addition, enzymatic assays were performed to investigate potential mechanisms underlying its larvicidal effect.

The major constituent identified in *P. marginatum* EO was 3',4'-methylenedioxypropiophenone, also known as 3,4-(methylenedioxy) phenyl-1-propanone (MDP1P), a phenylpropanoid commonly found in *Piper* species. This compound has also been reported as the principal component of *P. marginatum* EO specimens from the Amazon region (Da Silva et al. 2016; Gonçalves et al. 2019). Da Silva et al. (2016) described 3,4-methylenedioxypropiophenone (21.8%), elemol (5.9%), β-caryophyllene (5.0%), and 2-methoxy-4,5-methylenedioxypropiophenone (4.8%) in EOs obtained from leaves and dry stems collected in the Amazon forest. Similarly, Gonçalves et al. (2019) identified 3,4-methylenedioxypropiophenone (10.4%), bicyclogermacrene (10.2%), and germacrene D (9.9%) as major constituents. Here, the main compounds detected were 3',4'-methylenedioxypropiophenone (18.52%), β-caryophyllene (8.49%), γ-terpinene (8.05%), trans-β-ocimene (7.49%), and elemicin (6.07%).

Variations in EO composition are well documented and may be influenced by geographic origin, season of collection, temperature, light exposure, developmental stage, soil conditions, and harvest timing (de Morais 2009). Investigating *P. marginatum* populations from different ecosystems in the Brazilian Amazon, Andrade et al. (2008) described seven chemotypes (I–VII) based on differences in leaf oil composition. Chemotype III contained 3,4-methylenedioxypropiophenone (40.2%), myristicin (16.0%), (E)-β-ocimene (15.2%), γ-terpinene (14.4%), and β-caryophyllene (10.6%). Among the chemotypes described, chemotype III showed the greatest similarity to the oil characterized in our results.

The results demonstrated that *P. marginatum* EO exerted a concentration-dependent effect on both larval mortality and egg hatching inhibition in *Lu. longipalpis*. A marked reduction in egg hatching was observed at concentrations ≥ 1%, with higher concentrations inhibiting hatching by more than 50%. These findings indicate that the EO not only affects larval survival but also disrupts early developmental stages, with important implications for population control.

Although the highest concentration tested (4%, 40 mg/mL) exceeds those reported in some insecticidal studies, similar ranges have been employed in larvicidal assays using plant EOs against mosquito larvae. For example, *Vanillosmopsis arborea* EO showed significant larvicidal activity against *Ae. aegypti* larvae, with LC₅₀ values ranging from approximately 15 to 28 mg/mL (Furtado et al. 2005), supporting the biological relevance of EO concentrations in the tens of mg/mL range.

The ovicidal activity of *P. marginatum* EO against agricultural pests has previously been reported. Krinski et al. (2018) evaluating EOs from 18 Brazilian *Piper* species against eggs of *Anticarsia gemmatalis*, found that *P. marginatum* EO was among the most effective in preventing larval hatching, inducing mortality above 75% at 1% concentration (LC₅₀ = 1%; LC₉₀ = 2.3%). Guedes et al. (2020) assessed the effect of *P. marginatum* EO on embryonic development in eggs of *Spodoptera frugiperda* and reported an LC₅₀ of 152.95 ppm. In our experiments, LC₅₀ and LC₉₀ values for *Lu. longipalpis* eggs were 1.18% and 2.41%, respectively.

Sand fly eggs are generally considered the most resistant developmental stage to EO exposure, as reported by Maciel et al. (2010) who evaluated *Eucalyptus* spp. EOs against eggs, larvae, and adults of *Lu. longipalpis*. However, our findings indicate that *P. marginatum* EO exhibits substantial ovicidal activity at relatively low concentrations. The ovicidal effect of EOs has been attributed to the obstruction of gas exchange structures between the embryo and the external environment (e.g., the corium and micropyles), thereby disrupting embryonic development. Alternatively, bioactive compounds present in EOs may exert direct toxic effects that, combined with their physicochemical properties, contributing to egg mortality (Rajapakse and Senanayake 1997; Krinski et al. 2018).

The larvicidal activity of *P. marginatum* EO has also been reported for insects of medical importance, including *Ae. aegypti*. Moncayo-Baño et al. (2024) observed 100% larval mortality at 50 µL/mL against third- and fourth-instar *Ae. aegypti*. In *Lu. longipalpis*, Maciel et al. (2010) reported mortality rates above 70% in second-instar larvae exposed to *Eucalyptus staigeriana* EO at 4 mg/mL. In this study, 100% mortality of second-instar *Lu. longipalpis* larvae were observed at concentrations between 2 and 4%.

EOs from *Piper* species have been widely reported as effective natural pesticides against arthropods vectors. The EO of *P. capitarianum* exhibited larvicidal activity against *Ae. aegypti* and *Ae. albopictus* (LC₅₀ = 87.6 μg/mL and 76.1 μg/mL, respectively) (França et al. 2021). Similarly, *P. nigrum* EO demonstrated larvicidal activity against *Anopheles* spp., vectors of *Plasmodium* spp. (Samuel et al. 2016). In ticks, *P. aduncum* EO caused high mortality in *Amblyomma sculptum* larvae, along with reduced acetylcholinesterase activity (Pereira Filho et al. 2024).

Unlike cypermethrin, which primarily targets the nervous system through well-defined molecular mechanisms (Kaur and Singh 2021), EO represents a complex mixture of bioactive compounds that may act on multiple physiological pathways. This multi-target profile may explain the consistent yet potent biological effects observed across developmental stages, rather than the acute toxicity typically associated with synthetic insecticides.

Regarding the enzymatic assays, exposure of larvae to LC₅₀ and LC₉₀ concentrations of EO resulted in a non-significant trend toward reduced acetylcholinesterase activity. Although these findings do not provide direct evidence of acetylcholinesterase inhibition as the primary mechanism of action, they do not exclude the possibility of interference with cholinergic signaling. Importantly, the absence of a statistically significant inhibition suggests that additional molecular targets and detoxification pathways are likely involved in the observed larvicidal activity.

Taken together, these results demonstrate that *P. marginatum* EO exhibits biologically relevant insecticidal activity against immature stages of *Lu. longipalpis*. However, its mode of action appears to be mechanistically complex and potentially multifactorial. Further investigations assessing other detoxification enzymes and isolated major constituents of the EO will be essential to clarify its mechanism of action and to evaluate its potential as an alternative or complementary strategy for sand fly control.

## Conclusion

This study characterized the chemical composition of *P. marginatum* EO, identifying 3',4'-methylenedioxypropiophenone, a phenylpropanoid commonly found in plants of the genus *Piper* as its most abundant constituent. It also demonstrated the EO’s ovicidal and larvicidal activity against *Lu. longipalpis* under laboratory conditions. The EO induced a concentration-dependent reduction in egg hatching and larval survival, with complete ovicidal and larvicidal effects observed at higher concentrations.

These findings confirm the biological activity of *P. marginatum* EO against immature stages of *Lu. longipalpis* and provide original evidence supporting its potential use as an alternative control tool for this vector. Further studies are required to evaluate its mechanisms of action, optimize application strategies and assess its effectiveness and safety under field conditions.

## Data Availability

The authors confirm that all data generated or analyzed during this study are included in this published article.
